# Inter-Row Reflective Film Mulching Revealed the Regulation of Ground-Reflected Light on Grape Flavoromics

**DOI:** 10.3390/foods15050930

**Published:** 2026-03-06

**Authors:** Ning Shi, Hao-Cheng Lu, Meng-Bo Tian, Ming-Yu Li, Chang-Qing Duan, Jun Wang, Fei He

**Affiliations:** 1 Center for Viticulture and Enology, College of Food Science and Nutritional Engineering, China Agricultural University, Beijing 100083, China; wyyxsn@163.com (N.S.);; 2 Key Laboratory of Viticulture and Enology, Ministry of Agriculture and Rural Affairs, Beijing 100083, China

**Keywords:** reflective film, grape, flavonoids, aromas

## Abstract

Inter-row mulching with reflective film (RF) has been increasingly adopted in cool-climate vineyards to improve light availability and promote grape ripening. This study investigated the effects of ground-reflected light on the flavoromic profiles of wine grape berries (*Vitis vinifera* L.) over two consecutive vintages (2020–2021) in the Beijing Fangshan region of Eastern China, an area characterized by high precipitation and limited sunlight during ripening. Physicochemical analyses showed that RF treatment significantly increased total soluble solids (TSSs) and decreased titratable acidity (TA) at harvest. Targeted metabolomic analyses using HPLC–MS and GC–MS identified 21 flavonoids and 35 volatile compounds responsive to altered light conditions. RF treatment markedly enhanced the accumulation of anthocyanins and flavonols, especially malvidin-based derivatives, and increased terpene and norisoprenoid concentrations, while C_6_/C_9_ compounds were more abundant in control berries. Multivariate analysis revealed that PC1 was mainly associated with anthocyanin accumulation, clearly separating RF-treated samples, whereas PC2 reflected differences in flavonols and flavan-3-ols, with higher flavonols under RF and higher skin- and seed-derived flavan-3-ols in controls. Overall, these findings demonstrate that ground-reflected light plays a critical role in modulating grape flavor composition and provides practical guidance for improving fruit quality in suboptimal climatic regions.

## 1. Introduction

Wine, prized for its distinctive flavor, is widely appreciated by consumers worldwide. According to a report by the International Organisation of Vine and Wine (OIV), global wine production is projected to be between 228 mhl and 235 mhl by 2025 [1]. The color, aroma, and taste of wines are largely determined by the secondary metabolites accumulated in grape berries during ripening, including flavonoids and volatile compounds.

Among flavonoids, anthocyanins mainly accumulate in the skin of red grapes (apart from teinturier grapes, which synthesize anthocyanins both in skins and flesh) and determine the colors of red grapes and wines. Flavonols, found in grape skins, play a crucial role in the color stability of red wines through their copigmentation with anthocyanins [2] and provide a bitter taste to the wines. As the most abundant flavonoid compound in grape berries, found in the skins and seeds, or even stems, flavanols contribute to the bitterness and astringency of grapes and wines, as well as the copigmentation effect and synthesis of polymeric anthocyanins for the red wine color stability.

Although many aromatic compounds in wines are produced by yeast during fermentation or extracted from oak products during wine aging, many others are already present in grapes and fermented in unaltered forms or with only minor modifications. These aromatic compounds are mainly stored in a free or glycoside-bound state in the skin and flesh of grapes, determining the varietal aroma and characteristics of wines [3]. The grape-derived aromatic compounds mainly include terpenes, norisoprenoids, and C_6_/C_9_ compounds. Terpenes, the most important volatile compounds in grapes and wines, are largely responsible for floral and fruity aromas, with some monoterpenes possessing the most odoriferous [4]. Norisoprenoids are present in grapes only at trace levels, but most have very low sensory thresholds and are important sources of floral and fruity aromas in grapes and wines. The C_6_/C_9_ compounds, which mainly include C_6_/C_9_ aldehydes and alcohols, are characterized by a typical herbaceous aroma and are also referred to as green leaf volatiles [5].

The flavor compounds in grape berries were extremely sensitive to the changes in the environmental factors [6]. Therefore, many studies focused on regulating the microclimate in the cluster zone through viticulture measures to change the concentrations of flavor compounds in grape berries and wines [7]. Inter-row mulching treatment represents a specific ground management practice in viticulture. Depending on the mulch materials, it could regulate soil temperature, humidity, and microbiome, as well as the microclimate in the cluster zone, thereby adjusting grape and wine quality in a targeted manner [8]. Light-colored or reflective mulching materials such as white or silver reflective films, shells, gravels, and crushed glass could increase the photosynthetically active radiation reflected from the ground to the grape cluster zone [9,10,11]. Yuan et al. [12] found that covering reflective film on the ground significantly altered the berry weight and the sugar/acid ratio of ‘Shine Muscat’ grapes. However, Olejar et al. [13] reported that the white inter-row mulching had no significant impact on the grape yield, the number and weight of grape clusters, and the TA and pH of berries. There is also no consistent conclusion in studies on the impact of inter-row mulching treatment on flavonoid and aroma compounds. For flavonoid compounds, Tian et al. [11] indicated that light-colored gravel covering enhanced the flavonol concentrations of grape berries. Moreover, Osrecak et al. [14] found that the inter-row red reflective film covering increased the concentrations of (-)-epicatechin and gallic acid in ‘Merlot’, ‘Teran’, and ‘Plavac Mali’ wines. Another relatively early study showed that white or silver reflective film covering had almost no impact on the concentrations of total phenols, flavonols, and anthocyanins in grape berries [10]. As for aromatic compounds, Reynolds et al. [15] reported that inter-row mulching with reflective film could increase the concentrations of free and bound terpene compounds in ‘Riesling’ grape berries and reduce the herbaceous flavor of the wines. Tian et al. [11] found that light-colored gravel covering reduced the overall aroma of grapes. However, some studies showed that the white inter-row mulching treatment had no impact on the aroma profile of ‘Malbec’ wines [13]. In a nutshell, there were still controversies and deficiencies in the studies on the impact of inter-row mulching with reflective materials on the concentrations of flavor compounds in grape berries and wines, which might be closely related to the terroir conditions of different experimental sites, especially the sunlight.

Optimal accumulation of the flavor-related metabolites in grape berries required sufficient light exposure, which could be limited in humid, cloudy regions. Thus, inter-row mulching with reflective materials was mostly used in vineyards in cool regions, aiming to address the problem of berries not reaching optimal maturity. The Fangshan region of Beijing, located in Eastern China, is one of the country’s major wine regions. During the grape-growing season, this region usually faced significant environmental challenges, including high rainfall and low solar irradiance, often resulting in delayed or incomplete berry maturation. To address this issue, in the present study, inter-row mulching with reflective films was adopted as a canopy management strategy to increase the light reflection from the soil surface into the fruit zone, thereby enhancing the photoregulation of secondary metabolism. The systemic effects of ground-reflected light on grape flavoromics, especially flavonoid and aromatic compounds in commercially harvested grape berries between the treatment and control for two consecutive years, were evaluated. We hypothesized that inter-row mulching with reflective film would accelerate grape ripening and improve fruit quality by modulating key flavor-associated pathways. This study aimed to elucidate how reflected light regulated the accumulation of flavonoid and volatile compounds in grape berries, providing both scientific insights and practical guidance for sustainable viticulture in light-limited regions.

## 2. Materials and Methods

### 2.1. Vineyard, Experimental Design, and Sampling

This research was conducted in a commercial vineyard of Qianyuan Winery, Fangshan District, Beijing (39°78′ North, 116°06′ East), during the vintages of 2020 and 2021. The meteorological data (air temperature, precipitation, and relative humidity) of the vineyard during grape development were obtained from the nearest meteorological station (approximately 10.8 km away) of the China Meteorological Data Center (https://data.cma.cn/ (accessed on 6 May 2025)).

Uniform parcels of *Vitis vinifera* L. cv. ‘Cabernet Sauvignon’ (Vitis International Variety Catalogue, VIVC number 1929), ‘Cabernet Franc’ (VIVC number 1927), and ‘Marselan’ (VIVC number 16383) were selected. All grapevines were trained using modified vertical shoot positioning (M-VSP) and managed with locally consistent viticulture. The grapevines were planted on flat ground with rows oriented north–south, at a row and vine spacing of 3 m and 1 m, respectively. A complete block randomized design was used: reflective aluminum-coated polyethylene film (reflectivity > 85%; Nantong Lipeng New Material Co., Ltd., Haian, China) was laid between rows immediately after fruit set as the treatment (RF), while the control (CK) plots remained uncovered. A photograph of the vineyard with the reflecting film is shown in Supplementary Figure S1. For each cultivar, three biological replicates per treatment were employed, and each replicate included at least 50 vines.

When the total soluble solids of all treatment and control groups of each variety reached 15 °Brix, approximately 600 grape berries were randomly collected from each replicate. Fifty berries were subsampled randomly for the physicochemical analysis, while the remaining fruits were flash-frozen in liquid nitrogen and stored at −80 °C for the subsequent flavoromic determination.

### 2.2. Physicochemical Analysis of Grapes

The grape berries were weighed using an analytical balance (BSA223S, Sartorius, Göttingen, Lower Saxony, Germany), then placed in resealable plastic bags and manually pressed to obtain juice. The total soluble solids (TSSs) and pH values were detected using a digital refractometer (PAL-1, Atago, Tokyo, Japan) and a pH meter (Sartorius PB-10, Göttingen, Germany), respectively. The titratable acidity (TA) was determined by titration with 0.1 M NaOH to pH 8.2 and reported as g/L tartaric acid equivalent per liter according to the OIV compendium of methods [16].

### 2.3. The Extraction and Analysis of Flavonoids in Grapes

For each replicate, about 60.0 g of mature berries was used for flavonoid determination. Their skins and seeds were peeled off using a scalpel and forceps under liquid nitrogen, then pulverized separately into powder and freeze-dried at −40 °C. The extraction of flavonoids was consistent with the method described by Shi et al. [17]. All extracts were filtered through a 0.22 μm nylon membrane (MEMBRANA, Wuppertal, North Rhine-Westphalia, Germany) and analyzed using high-performance liquid chromatography/triple-quadrupole tandem mass spectrometry (HPLC-QqQ-MS/MS). An Agilent 1200 series HPLC system equipped with an Agilent 6410 QqQ instrument (Agilent Technologies Inc., Palo Alto, CA, USA) was used. The column was Poroshell 120 EC-C_18_. The detailed procedures of HPLC and MS conditions have been described previously [17]. Briefly, the mobile phase flow rate was 0.4 mL/min, and the column oven temperature was set at 55 °C. Mass spectrometry was performed using an AJESI ion source with a spray voltage of 4 kV, an ion source temperature of 150 °C, a drying gas temperature of 350 °C, a flow rate of 12 L/h, and a nebulizer pressure of 35 psi. The detector had a multiple reaction monitoring (MRM) mode. The mobile phases used for elution are 0.1% aqueous formic acid solution as phase A, and 50/50 methanol–acetonitrile solution containing 0.1% formic acid as phase B. The mobile phase flow rate was 0.4 mL/min. The elution time was 32 min with the following gradient: 0–28 min, 10–46% B; 282–32 min, 46–10% B. Following elution, the column was washed with 10% B for 5 min for equilibration. Flavonoid compounds were quantified using a calibration curve: anthocyanins were quantified using malvidin-3-*O*-glucoside, flavonols with quercetin-3-*O*-glucoside, and flavanols with (+)-catechin, (-)-epicatechin, (-)-epicatechin-3-*O*-gallate, and (-)-epigallocatechin. All flavonoid compounds in grapes were expressed as μg/kg fresh weight (FW).

### 2.4. The Extraction and Analysis of Volatiles in Grapes

For each replicate, about 60 g of berries were meticulously deseeded and ground into powder under liquid nitrogen with the addition of 1.0 g of polyvinylpyrrolidone (Sigma-Aldrich, Carlsbad, CA, USA) and 0.5 g of D-gluconic acid lactone (Sangon Biotech, Shanghai, China). This powder melted at 4 °C for 4 h and then centrifuged at 8000× *g* for 10 min to obtain a clear juice, which could be used directly for the determination of free-form volatiles. The extraction of bound volatile compounds of grapes was according to Shi et al. [17]. Briefly, 2 mL of clear juice was added to the solid-phase extraction column (Cleanert PEP, 1000 mg/12 mL) activated with 10 mL of methanol and 10 mL of water, followed by sequential elution with 2 mL of water to remove low-molecular-weight polarized sugars and 2 mL of dichloromethane to remove free aroma substances, Finally, 20 mL of methanol was used and collected, and the flow rate of the elution process was maintained at 2 mL per minute. The final collected eluate was concentrated to dryness by a rotary evaporator under vacuum at 30 °C and then redissolved in 10 mL of citrate/sodium citrate buffer solution (0.2 M, pH = 5). The enzymatic hydrolysis of glycosidic precursors was conducted at 40 °C for 16 h by adding 100 μL AR 2000 (Rapidase, 100 g/L, DSM Food Specialties, Seclin, France). The solution was used for the subsequent extraction and detection of bound volatile compounds. Next, volatile compounds were determined according to Shi et al. [17]. Then, 5 mL of juice/solution was added to a 20 mL vial containing 1 g NaCl and 10 µL of internal standard (4-methyl-2-pentanol) and concentrated using HS-SPME. The volatile compounds in grapes were determined using an Agilent 6890 gas chromatography (GC) coupled with an Agilent 5973 mass spectrometer (MS) with an HP-INNOWAX capillary column (60 m × 0.25 mm, 0.25 μm). The volatile compounds were identified by matching retention indices (RIs) and mass spectra with reference standards in the NIST 14 MS library. Target analytes included C_6_/C_9_ compounds (e.g., hexanal, (*Z*)-3-hexenol), monoterpenes (e.g., linalool, geraniol), norisoprenoids (e.g., *β*-damascenone, TDN), and esters. Quantification of volatile compounds was based on the calibration curves of volatile standards. Semi-quantification was conducted for volatile compounds with no corresponding standards. These compounds were quantified using internal standard curves of standards with similar chemical structures, functional groups, and/or similar carbon numbers. All volatile compounds in grapes were expressed as μg/kg fresh weight (FW).

### 2.5. Statistical Analysis

The Student’s *t*-test and multi-way analysis of variance (ANOVA) were performed using SPSS 26.0 to confirm the significance of the differences between varieties at a significant level of *p* < 0.05. The figures were drawn using GraphPad Prism 8.0.2. Principal component analysis (PCA) and orthogonal partial least squares discriminant analysis (OPLS-DA) were performed using Simca 14.1.

## 3. Results

### 3.1. Meteorological Data

As shown in Table 1, the highest average monthly temperatures occurred from June to August in both 2020 and 2021, at 26.9 °C and 26.7 °C, respectively. The average monthly maximum temperature was 32.7 °C in June 2020, while the average monthly minimum temperature was 14.0 °C in May 2021. Compared to climate data from the past decade, temperatures were lower in May and July of 2020 and 2021. Conversely, June was warmer in 2020, and August was cooler in 2021. The total precipitation during the growing seasons was 430.0 mm and 573.0 mm in 2020 and 2021, respectively, with most of the rainfall concentrated between July and September. Notably, precipitation in July and September 2021 was significantly higher than in 2020, resulting in increased relative humidity. In addition, precipitation in June of both 2020 and 2021 was below the average for the past decade, while precipitation in August and September was higher. Meanwhile, precipitation in July was lower in 2020 and higher in 2021 than the average for the past decade. Meteorological factors had a critical influence on grape berry ripening and quality. While temperature variations between the two years were minimal, differences in precipitation and the associated changes in sunshine likely contributed to variations in the flavor composition of grape berries.

### 3.2. Physicochemical Indicators of Grape Berries

**Figure 1:**
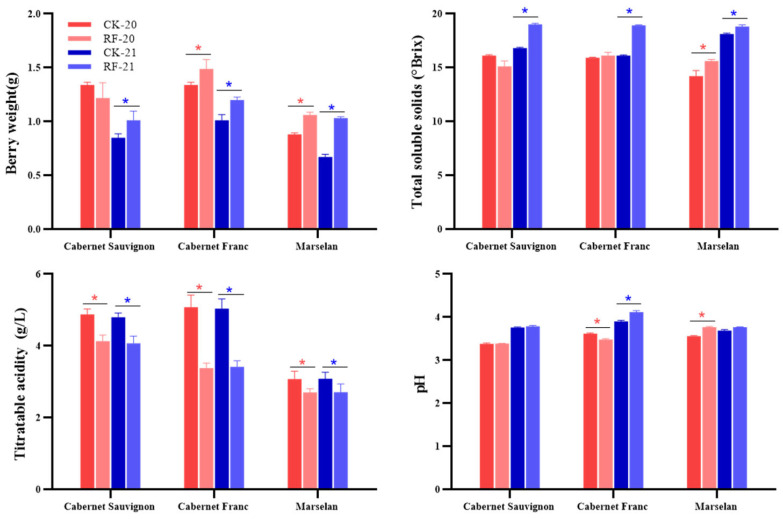
The physicochemical indicators of grape berries under RF treatment in two years. The * indicates significant differences between RF treatment and CK based on Student’s test, *p* < 0.05.

The physicochemical indicators of grape berries are shown in Figure 1. The differences were significant based on Student’s *t*-test (*p* < 0.05). Berry weight in the RF treatment was significantly higher than CK over the two years, except for ‘Cabernet Sauvignon’ in 2020. The total soluble solids (TSS) of grapes showed that RF grapes had significantly higher TSS levels in 2021, while in 2020, RF only significantly increased the TSS level of ‘Marselan’ grapes. Titratable acidity (TA) was consistently and significantly reduced using RF in all three grape cultivars over the two years. pH was less affected by RF. The pH of ‘Marselan’ grapes was significantly increased using RF in 2020. However, the effect of RF on the pH of ‘Cabernet Franc’ grapes was inconsistent between the two years, with RF significantly reducing pH in 2020 and the opposite in 2021. Overall, RF treatment significantly improved grape maturity at harvest. Berries from RF-treated vines exhibited higher TSS and lower TA compared to CK samples, indicating accelerated sugar accumulation and acid degradation during ripening.

### 3.3. The Flavonoids of Grape Berries

**Figure 2:**
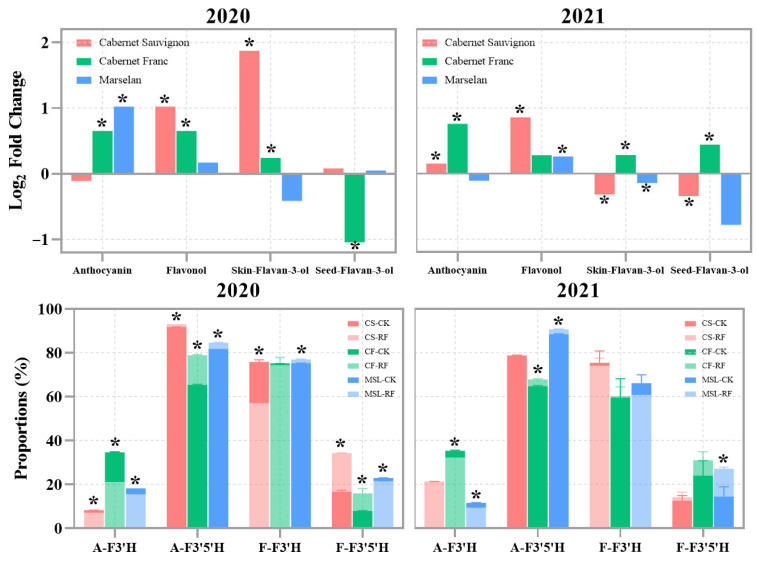
Effect of RF treatment on the flavonoid composition and the proportion of metabolites from different pathways. Log_2_ fold changes represent the changes in metabolite concentrations between RF treatment and CK. The proportion is the ratio of each classified anthocyanin or flavonol to the total anthocyanin or flavonol. The * indicates significant differences between RF treatment and CK based on Student’s test, *p* < 0.05.

The overall effect of RF treatment on flavonoid profiles in grape berries is shown in Figure 2. To better highlight the effects of RF treatment, log2 fold changes between RF and CK were used. The differences were significant based on Student’s *t*-test (*p* < 0.05). In 2020, RF treatment significantly increased flavonol and skin-flavan-3-ol levels in ‘Cabernet Sauvignon’, as well as anthocyanin, flavonol, and skin-flavan-3-ol levels in ‘Cabernet Franc’, and anthocyanin levels in ‘Marselan’, while reducing seed-flavan-3-ol levels in ‘Cabernet Franc’. In 2021, RF treatment significantly increased anthocyanin and flavonol levels in ‘Cabernet Sauvignon’, anthocyanin and skin-flavan-3-ol levels in ‘Cabernet Franc’, and flavonol levels in ‘Marselan’, but decreased skin- and seed-flavan-3-ol levels in ‘Cabernet Sauvignon’ and skin-flavan-3-ol levels in ‘Marselan’. Overall, RF treatment promoted anthocyanin and flavonol accumulation in grape berries, though the effects on flavan-3-ols were inconsistent across years.

Flavonoid-3′-hydroxylase (F3′H) and flavonoid-3′,5′-hydroxylase (F3′5′H) in the flavonoid pathway competitively regulate flavonoid biosynthesis. F3′H was involved in producing cyanidin-based anthocyanins, quercetin- and isorhamnetin-based flavonols, while F3′5′H was responsible for synthesizing delphinidin-based anthocyanins and myricetin-, laricitrin-, and syringetin-based flavonols. Based on these metabolic pathways, we classified various types of anthocyanins and flavonols into four categories: anthocyanin-F3′H (A-F3′H), A-F3′5′H, flavonol-F3′H (F-F3′H), and F-F3′5′H. In 2020, RF treatment significantly increased the proportions of A-F3′5′H and F-F3′5′H in ‘Cabernet Sauvignon’ and ‘Cabernet Franc’, as well as A-F3′5′H and F-F3′H in ‘Marselan’. Conversely, RF treatment decreased the proportions of A-F3′H and F-F3′H in ‘Cabernet Sauvignon’, A-F3′H in ‘Cabernet Franc’, and A-F3′H and F-F3′5′H in ‘Marselan’. In 2021, RF treatment again significantly increased the proportions of A-F3′5′H in ‘Cabernet Franc’ and both A-F3′5′H and F-F3′5′H in ‘Marselan’, while reducing the proportions of A-F3′H in both ‘Cabernet Franc’ and ‘Marselan’. Although the effects in 2021 were less pronounced than in 2020, the overall trends were consistent between the two years. These results suggested that RF treatment enhanced flow through the F3′5′H pathway while reducing flow through the F3′H pathway, highlighting the competitive relationship between these two pathways.

### 3.4. Photosensitive Flavonoids of Grape Berries

**Figure 3:**
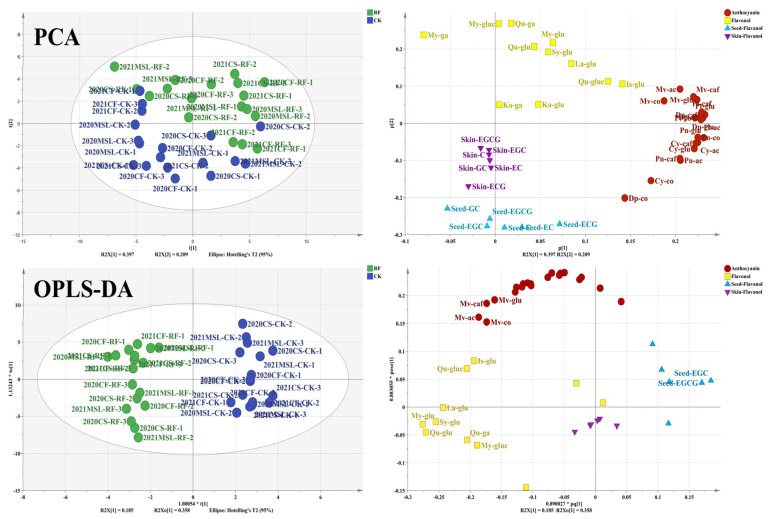
PCA and OPLS-DA based on the flavonoid compounds of grape berries. The data on flavonoid concentrations were standardized for each cultivar and year. The loading plot of OPLS-DA hid the labels of flavonoids with VIP score of less than 1.0. glu, glucoside; ac, acetylglucoside; co, coumaroylglucoside; caf, caffeoylglucoside; gla, galactoside; gluc, glucuronide; Cy, cyanidin; Dp, delphinidin; Pn, peonidin; Pt, petunidin; Mv, malvidin; My, myricetin; Qu, quercetin; Ka, kaempferol; Sy, syringetin; Is, isorhamnetin; La, laricitrin; C, catechin; EC, epicatechin; GC, gallic acid; EGC, epigallocatechin; ECG, epicatechin gallate; EGCG, epigallocatechin gallate.

In total, 37 flavonoid compounds were identified across all grape samples, comprising 20 anthocyanins, 11 flavonols, and 6 flavan-3-ols, as detailed in Supplementary Table S1. To eliminate the influence of year and cultivar differences, flavonoid concentrations were standardized for each cultivar and year. Principal component analysis (PCA) was then applied to distinguish samples from RF treatment and CK (as shown in Figure 3). The first principal component (PC1), accounting for 39.7% of the total variance, was primarily associated with anthocyanins. Most RF-treated samples clustered in the positive region of PC1, suggesting that RF treatment led to higher anthocyanin concentrations in grape berries. The second principal component (PC2), explaining 20.9% of the variance, was dominated by flavonols and flavan-3-ols. RF-treated samples generally showed higher flavonol concentrations and were positioned in the positive region of PC2, while CK samples, with higher concentrations of skin- and seed-flavan-3-ols, were found in the negative region of PC2. Further screening using orthogonal partial least squares discriminant analysis (OPLS-DA) identified flavonoids with a VIP score greater than 1.0. The results revealed that malvidin-based anthocyanins, most flavonols (excluding kaempferol), and seed-EGC and seed-EGCG contributed most significantly to the differences between RF and CK. The differences between RF and CK were mainly caused by light, indicating that these compounds were likely photosensitive flavonoids. Specifically, malvidin-based anthocyanins and most flavonols were upregulated in grape berries under higher light levels, whereas seed-EGC and seed-EGCG concentrations were reduced. Thus, a total of 21 photosensitive flavonoid compounds were identified, all of which responded to changes in light intensity. Overall, RF promoted the accumulation of anthocyanins and flavonols across cultivars. Malvidin-based anthocyanins and most flavonols were upregulated under higher light conditions, particularly in ‘Cabernet Sauvignon’ and ‘Marselan’. Although the magnitude of change was less pronounced in 2021 than in 2020, the overall trends remained consistent across years.

### 3.5. The Volatiles of Grape Berries

**Figure 4:**
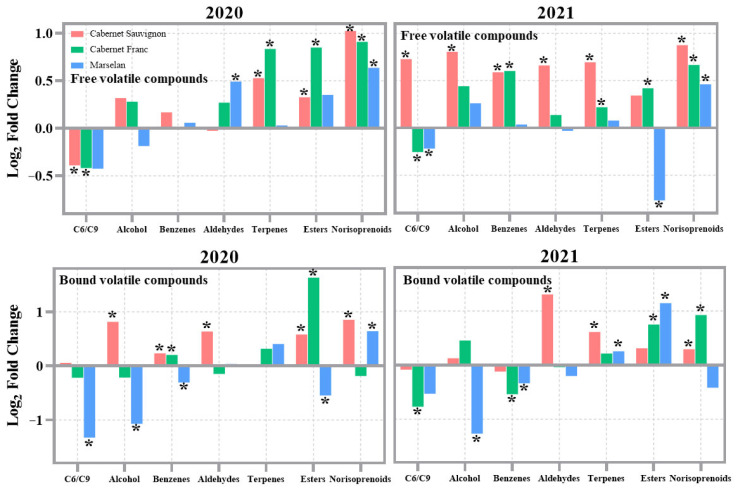
Effect of RF treatment on the volatile compounds of grape berries in 2020 and 2021. Log_2_ fold changes represented the changes in metabolite concentrations between RF treatment and CK. The * indicates significant differences between RF treatment and CK based on Student’s test, *p* < 0.05.

In this study, volatile compounds in grape berries were classified into seven categories based on their chemical structure: C_6_/C_9_ compounds, alcohols, benzenes, aldehydes, terpenes, esters, and norisoprenoids. Figure 4 illustrates the effect of RF treatment on the total concentrations of each type of volatile compound. The differences were significant based on Student’s *t*-test (*p* < 0.05). For free volatile compounds, RF treatment significantly increased the concentrations of aldehydes and norisoprenoids in ‘Marselan’, as well as terpenes, esters, and norisoprenoids in’ Cabernet Sauvignon’ and ‘Cabernet Franc’ in 2020. In contrast, RF treatment notably reduced the concentrations of C_6_/C_9_ compounds in both ‘Cabernet Sauvignon’ and ‘Cabernet Franc’ in the same year. In 2021, RF treatment significantly increased the concentrations of C_6_/C_9_ compounds, alcohols, benzenes, aldehydes, terpenes, and norisoprenoids in ‘Cabernet Sauvignon’, benzenes, esters, and norisoprenoids in ‘Cabernet Franc’, as well as norisoprenoids in Marselan. However, it reduced the concentrations of C_6_/C_9_ compounds in ‘Cabernet Franc’ and both C_6_/C_9_ compounds and esters in ‘Marselan’. For bound volatile compounds, RF treatment in 2020 significantly increased the concentrations of alcohols, benzenes, aldehydes, esters, and norisoprenoids in ‘Cabernet Sauvignon’, benzenes and esters in ‘Cabernet Franc’, and norisoprenoids in ‘Marselan’. Conversely, it decreased the concentrations of C_6_/C_9_ compounds, alcohols, benzenes, and esters in ‘Marselan’. In 2021, RF treatment significantly increased the concentrations of aldehydes, terpenes, and norisoprenoids in ‘Cabernet Sauvignon’, esters and norisoprenoids in ‘Cabernet Franc’, terpenes and esters in Marselan, while reducing the concentrations of C_6_/C_9_ compounds and benzenes in ‘Cabernet Franc’, as well as alcohols and benzenes in ‘Marselan’. The results from the two-year study demonstrated that RF treatments effectively increased the concentrations of terpenes, esters, and norisoprenoids, while reducing the levels of C_6_/C_9_ compounds in grape berries.

### 3.6. Photosensitive Volatiles of Grape Berries

**Figure 5:**
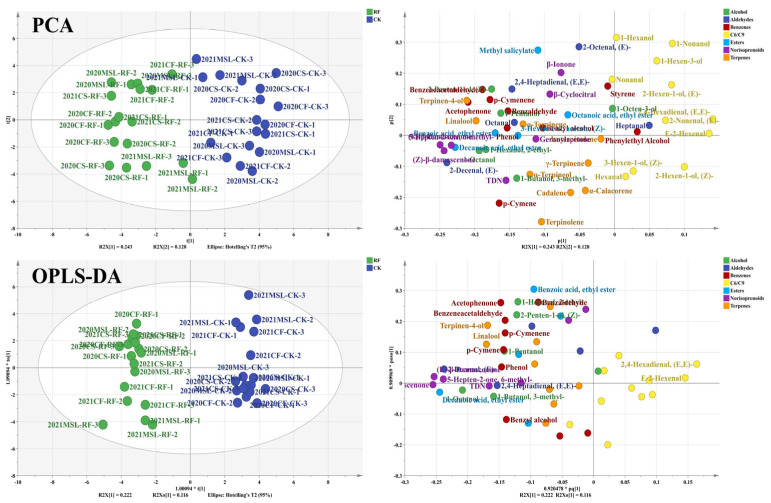
PCA and OPLS-DA based on the free volatile compounds of grape berries. The data of flavonoid concentrations were standardized for each cultivar and year. The loading plot of OPLS-DA hid the labels of flavonoids with VIP score less than 1.0.

A total of 52 free volatile compounds were detected across all grape samples, as listed in Supplementary Table S2. PCA and OPLS-DA were performed using their concentration data standardized for each cultivar and year (Figure 5). The PCA results clearly distinguish RF-treated grapes from the CK, with RF samples located in the negative part of PC1 and CK samples in the positive part. The PCA loading plot illustrated that the concentrations of C_6_/C_9_ compounds were higher in CK grapes, while the levels of other volatile compounds were higher in RF-treated grapes. Additionally, OPLS-DA was used to identify photosensitive free volatile compounds in grape berries. The results showed that 22 free volatile compounds, such as (*Z*)-*β*-damascenone, 1-octanol, (*E*)-2-decenal, were upregulated under higher light levels, while (*E*)-2-hexenal and (*E*, *E*)-2, 4-hexadienal were downregulated.

**Figure 6:**
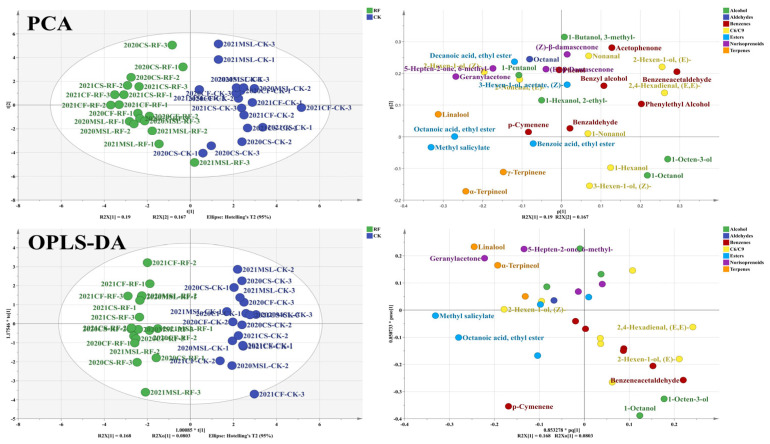
PCA and OPLS-DA based on the bound volatile compounds of grape berries. The data of flavonoid concentrations were standardized for each cultivar and year. The loading plot of OPLS-DA hid the labels of flavonoids with VIP score less than 1.0.

A total of 33 bound volatile compounds were identified across all grape samples, as detailed in Supplementary Table S3. PCA and OPLS-DA were performed based on their concentration data, which were standardized for each cultivar and year (Figure 6). The PCA results clearly distinguished RF treatment from CK, with RF located in the negative part of PC1 and CK in the positive part of PC1. The PCA loading plot illustrates that the concentrations of norisoprenoids, esters, and terpenes were higher in RF treatment, whereas the concentrations of alcohols, C_6_/C_9_ compounds, and benzenes were higher in CK. The OPLS-DA further identified 13 photosensitive bound volatile compounds. Among these, methyl salicylate, ethyl octanoate, p-cymenene, (*Z*)-2-hexen-1-ol, geranylacetone, linalool, α-terpineol, and 6-methyl-5-hepten-2-one were upregulated under higher light levels, while benzeneacetaldehyde, 1-octanol, 1-octen-3-ol, (*E*)-2-hexen-1-ol, and (*E*, *E*)-2,4-hexadienal were downregulated.

Thus, 35 compounds were found to be responsive to light modulation. RF treatment significantly enhanced the concentrations of terpenes and norisoprenoids—key contributors to floral and fruity aromas—in grape berries. In contrast, the levels of C_6_/C_9_ compounds—associated with green, grassy notes—were higher in control samples, indicating delayed senescence-related lipid oxidation in shaded conditions.

## 4. Discussion

The microclimate in the grape cluster zone, including factors such as temperature, humidity, and light, plays a crucial role in determining grape berry quality. Inter-row mulching with reflective film could significantly increase the light intensity in the cluster zone. Yuan et al. [12] showed that covering reflective film on the ground could double the amount of reflected light under the grapevine canopy compared to the control. It should be noted that although light intensity is generally associated with temperature, microclimatic factors such as cluster zone temperature were not monitored in the present study. Previous studies have reported that reflective film does not significantly affect the temperature [18,19]. Given that the experimental site is in a relatively cool region with limited solar radiation, the observed changes in grape flavor compounds are more likely attributable to modifications in the light environment rather than to thermal stress. Therefore, this study sought to explore the impact of light enhancement caused by reflective film on grape quality and establish a foundational framework for its application in viticulture.

In this study, grape physicochemical properties were significantly influenced by the application of reflective film (RF). The increased levels of reflected light accelerated berry ripening, enhanced the concentration of soluble solids, increased berry weight, and reduced the levels of titratable acid. The development of grape berries was closely related to the photosynthesis of leaves. A previous study showed that covering reflective film promotes the photosynthetic rates of grapevine [12]. This process typically resulted in higher sugar accumulation and reduced titratable acidity in ripening grape berries [11]. Among various physicochemical parameters, organic acids appeared to be the most responsive to environmental conditions [20], which might explain the most significant reduction in TA observed with RF treatment in this study. Both total acidity and pH value can reflect wine acidity to some extent, but their trends differ in this article. This may be due to concentration differences of different organic acids, as they have different dissociation abilities. Total acidity measures all acids that can be neutralized, while pH value measures the activity of free hydrogen ions. In addition, a correlation between grape berry weight and light environment was also reported [21]. Previous studies showed that grape berries exposed to higher light exhibit significantly higher weights [12,22], which were consistent with the current study.

Flavonoids were important flavor compounds in grapes and wines. Anthocyanins were the primary compounds responsible for the color of red grapes and wines. Flavonols protected grape berries from UV-B damage and served as co-pigments, enhancing wine coloration. Many studies demonstrated that the accumulation of anthocyanins and flavonols was particularly sensitive to changes in light conditions [23,24]. Previous studies showed that increased light intensity significantly upregulated key genes in the flavonol biosynthesis pathway, leading to higher flavonol concentrations in berries [25]. Conversely, shading treatments downregulated the expression of these genes and significantly inhibited flavonol accumulation [26]. These results highlight the regulatory role of light in flavonoid metabolism, promoting the synthesis of anthocyanins and flavonols, consistent with our study. Furthermore, the composition of anthocyanins and flavonols is linked to the expression levels of the F3′5′H and F3′H genes in this pathway [27]. Azuma et al. [25] reported that sufficient sunlight upregulates the F3′5′H gene, increasing the proportion of delphinidin-based anthocyanins in berries. Similarly, our study highlighted that light enhanced the synthesis of flavonoid compounds through the F3′5′H pathway, including malvidin-based anthocyanins and myricetin-, laricitrin-, and syringetin-based flavonols. Flavan-3-ols, the most abundant flavonoids in grape skins and seeds, contribute to the bitterness and astringency of grapes and wines [28]. However, the total concentration of flavan-3-ols in the RF treatment and CK showed no consistent differences over two years. This may indicate that light is not a direct factor affecting the accumulation of flavan-3-ols in grapes.

Volatile compounds played a crucial role in determining grape and wine quality due to their aromatic contributions. Terpenes were one of the most important aroma compounds that contributed floral and fruity notes to grapes and wines. Song et al. [29] reported that sunlight exposure and ultraviolet increased nerol, geraniol, and citronellol in Pinot Noir grapes. High light exposure has been shown to upregulate the expression of key genes involved in terpenoid metabolism, enhancing terpene accumulation in grape berries [30,31]. Norisoprenoids contribute substantially to the floral and fruity aroma of grape berries and wines [32]. Their accumulation is positively associated with sunlight exposure [33]. Young et al. [34] reported that high light contributes to the accumulation of norisoprenoids in grape berries due to elevated carotenoid concentrations. Carotenoids are precursors for the synthesis of norisoprenoids, and their concentration in red grapes is generally lower than that in white grape varieties. Joubert et al. [35] indicated that berries employed carotenoids and the associated xanthophyll cycles to acclimate to high light exposure. In contrast, C6/C9 compounds, which are commonly associated with herbaceous aromas, tended to decline in RF-treated berries. This reduction may reflect accelerated grape ripening under improved light conditions. These results indicated that RF treatment helped improve the aroma quality of grape berries.

While year-to-year variation was observed, possibly due to weather fluctuations, the consistency in directional trends underscored the robustness of light-mediated metabolic responses.

## 5. Conclusions

This study examined the impact of reflective film (RF) applied to the ground on the flavor profiles of three grape cultivars (‘Cabernet Sauvignon’, ‘Cabernet Franc’, and ‘Marselan’) over two years (2020–2021). RF treatment significantly enhanced TSS, increased berry weight, and reduced TA. Meanwhile, RF treatment promoted the accumulation of anthocyanins, flavonols, terpenes, and norisoprenoids in grape berries, while reducing the concentrations of seed-flavan-3-ols and C_6_/C_9_ compounds. Based on these changes in flavor compounds, RF appeared to be effective in modifying the light microclimate by reflecting sunlight into the cluster zone, thereby affecting berry flavor profiles and suggesting an important role of light availability in shaping grape quality. The OPLS-DA identified some photosensitive flavor compounds. The light-promoted compounds, such as malvidin-based anthocyanins, *β*-damascenone, and linalool, were found in higher concentrations under high-light conditions. Conversely, light-inhibited compounds, such as seed-EGC, (*E*, *E*)-2, 4-hexadienal, and (*E*)-2-hexenal, showed reduced concentrations with high light exposure. Overall, RF treatments had a positive effect on grape berry quality. These findings provided valuable recommendations for viticulturists in low-light regions and offered new insights into the role of light in shaping the flavor profiles of grapes. This practice offers a promising agronomic solution for overcoming light deficiency in humid, cloudy viticultural regions like Eastern China.

## Figures and Tables

**Table 1:** Meteorological data during the 2020 and 2021 growing seasons.

Month	T-Max (°C)		T-Min (°C)		T-Mean (°C)			RH (%)		PRCP (mm)		
	2020	2021	2020	2021	2020	2021	Average (2011–2020)	2020	2021	2020	2021	Average (2011–2020)
May	27.0	26.6	15.5	14.0	21.1	20.6	22.0	53.1	43.0	49.3	16.3	31.7
June	32.7	31.4	21.2	19.7	26.9	25.7	25.7	49.8	54.0	33.7	36.5	72.8
July	31.5	31.0	22.2	23.3	26.7	26.7	27.5	66.5	78.2	108.5	238.9	192.4
August	31.2	30.3	22.5	21.5	26.7	25.7	26.7	72.0	70.4	167.4	141.5	116.0
September	27.2	26.4	17	18.4	21.8	21.9	21.55	62.5	78.1	71.1	139.8	61.64

Note: T-Mean, average monthly temperature; T-Max, average monthly maximum temperature; T-Min, average monthly minimum temperature; RH, relative humidity; PRCP, precipitation.

## Data Availability

The original contributions presented in this study are included in the article/Supplementary Material. Further inquiries can be directed to the corresponding authors.

## Supplementary Materials

The following supporting information can be downloaded at https://www.mdpi.com/article/10.3390/foods15050930/s1, Figure S1: The photography of the vineyard with the reflecting film treatment; Table S1: The concentrations of flavonoid compounds in grape berries (μg/kg FW); Table S2: The concentrations of free volatile compounds in grape berries (μg/kg FW); Table S3: The concentrations of bound volatile compounds in grape berries (μg/kg FW).
