# Supplementary figures and images for: Inter-Row Reflective Film Mulching Revealed the Regulation of Ground-Reflected Light on Grape Flavoromics
Source: Foods. 2026 Mar 6;15(5):930. doi: 10.3390/foods15050930 (PMC12985225; doi:10.3390/foods15050930)

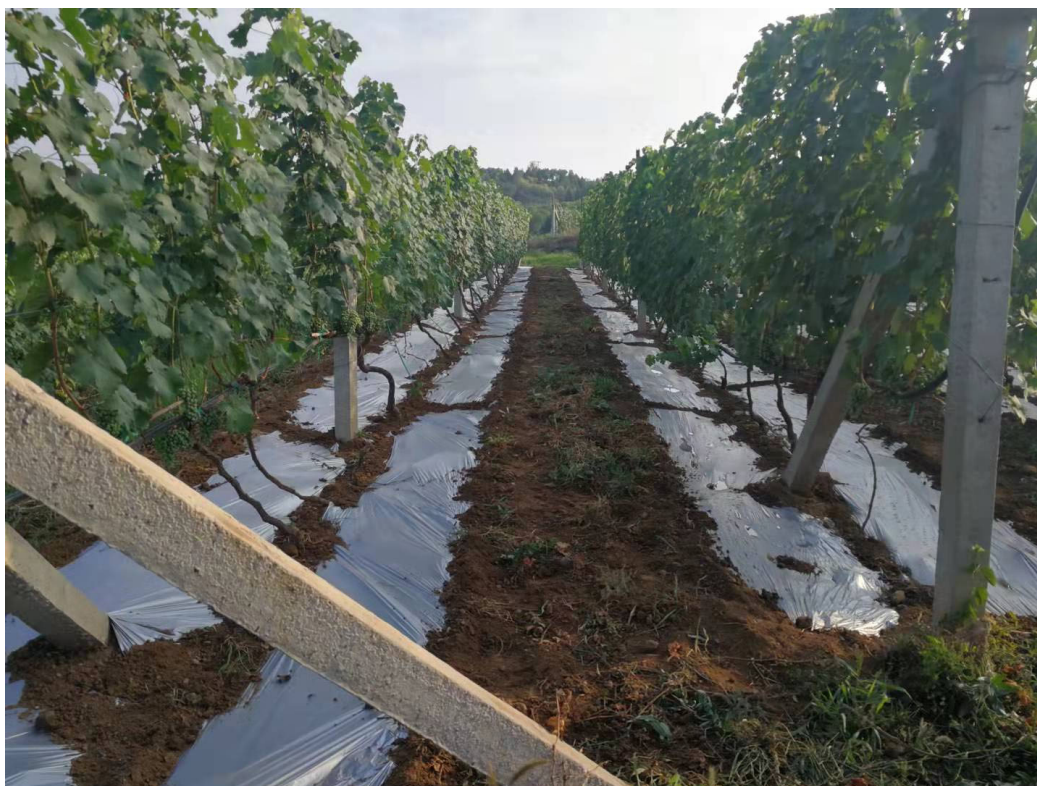

**Supplementary Figure 1.** The photography of the vineyard with the reflecting film treatment.

Supplement: Supplementary file 1 [file foods-15-00930-s001.zip › Supplementary Figure.pdf]
